# Ethanol sclerotherapy for management of endometriomas: an overview of systematic reviews

**DOI:** 10.3389/fendo.2025.1612899

**Published:** 2025-11-18

**Authors:** Johnny S. Younis, Nora Shapso, Ido Izhaki, Hugh S. Taylor

**Affiliations:** 1 Reproductive Medicine, Department of Obstetrics and Gynecology, Tzafon Medical Center, Poriya, Israel; 2 Azrieili Faculty of Medicine in Galilee, Bar-Ilan University, Safed, Israel; 3 Department of Evolutionary and Environmental Biology, University of Haifa, Haifa, Israel; 4 Department of Obstetrics, Gynecology and Reproductive Sciences, Yale School of Medicine, New Haven, CT, United States

**Keywords:** endometriosis, endometrioma, ethanol sclerotherapy, ovarian reserve, endometriotic cystectomy, overview, systematic reviews

## Abstract

**Background:**

Evidence suggests that endometrioma cystectomy can significantly reduce ovarian reserve. Ethanol sclerotherapy is a promising, minimally invasive alternative. This overview aims to critically evaluate systematic reviews that addressed the safety and efficacy of ethanol sclerotherapy in women with endometrioma and compared it to endometriotic cystectomy.

**Methods:**

A systematic search was conducted on PubMed, Medline, Epistemonikos, the Cochrane Library, and PROSPERO using the search terms “endometriosis OR endometrioma AND sclerotherapy.” Key outcomes assessed included adverse events, technical efficacy, pain relief, endometrioma recurrence, impact on ovarian reserve, ART success, and pregnancy outcomes. Two independent reviewers screened, reviewed, and selected relevant publications. They also evaluated the quality of the included systematic reviews using the modified version of the Assessing the Methodological Quality of Systematic Reviews (AMSTAR2) tool. Furthermore, they assessed the strength of evidence for the primary outcome measures according to the Grading of Recommendations Assessment, Development, and Evaluation (GRADE) system. The study protocol was registered in advance at PROSPERO under registration number CRD42024595209 on 10 October 2024.

**Results:**

Nine systematic reviews were eligible, analyzing between 386 and 1,642 procedures. The reviews reported high technical efficacy (95.0%-98.3%) and low adverse events (11.0%-12.0% minor, 1.0%-1.7% major). Outcomes for pain relief, ovarian reserve, and pregnancy rates were generally favorable. Pregnancy rates for ethanol sclerotherapy and endometriotic cystectomy appeared comparable; however, sclerotherapy resulted in larger number of eggs retrieved and no loss of ovarian reserve. Overall, the strength of evidence for sclerotherapy was low to very low. though larger sample sizes supported findings on technical efficacy and adverse events.

**Conclusions:**

Ethanol sclerotherapy is a viable alternative to endometriotic cystectomy for treating endometrioma in reproductive-age women. It has the advantage of being safe and effective for pain relief and potentially superior to cystectomy in preserving ovarian reserve. Future studies should evaluate sclerotherapy compared to cystectomy and expectant management in randomized controlled trials.

**Systematic Review Registration:**

https://www.crd.york.ac.uk/prospero/, identifier CRD42024595209.

## 1 Introduction

Endometriomas are a common and distinctive feature of endometriosis in women of reproductive age (1). Currently, the primary method of diagnosis is ultrasonography; when performed by skilled personnel, ultrasound eliminates the need for endoscopy or surgery for diagnosis (2). There has been an ongoing debate about the best approach to treating endometrioma, especially in women experiencing endometriosis-related pain and infertility (3, 4). Generally, management options include expectant, medical, surgical, or assisted reproductive technologies. The most appropriate management approach takes into consideration the woman’s age, ovarian reserve, symptoms, stage and sub-type of the disease, as well as her desire for childbirth.

There is compelling evidence today suggesting that endometrioma cystectomy using the stripping technique can lead to a significant and irreversible decrease in ovarian reserve (5–7). A recent systematic review and meta-analysis has shown that endometrioma cystectomy can result in a notable and irreversible decline in serum AMH levels by 39% and 57% in unilateral and bilateral cases, respectively, at 9–12 months after surgery (6). These functional findings, supported by histological studies (8–10), indicate that this procedure may limit reproductive lifespan and lead to early menopause (11–13).

To avoid these complications, other minimally invasive approaches to endometrioma management have been developed to minimize inadvertent operative damage to the ovarian reserve. These methods include endometrioma sclerotherapy, ablation using bipolar electrocoagulation or plasma energy, and laser vaporization. Unfortunately, these may still impact ovarian reserve if the energy reaches normal ovarian tissue. Ultrasound-guided sclerotherapy of endometriomas, mainly using ethanol, has become popular in recent years, with many published studies. Several systematic reviews have also been conducted to summarize existing experiences and guide management approaches, but their findings have been inconsistent and contradictory. For example, one review indicated that sclerotherapy leads to a lower recurrence rate of endometriomas and a better clinical pregnancy rate (14), while another review showed comparable results (15).

Systematic reviews and meta-analyses are essential for combining existing scientific information, strengthening the credibility of primary study findings, and pinpointing areas for further research (16). Furthermore, they can enhance the accuracy of evidence, as numerous studies are not large enough to yield definitive results. Nevertheless, unquestioningly adopting the findings of a single systematic review may carry several undisclosed risks (17).

The purpose of this study is to critically explore eligible systematic reviews that evaluated the safety and effectiveness of sclerotherapy, specifically with ethanol, in the management of women with endometrioma during their reproductive years. Specifically, we examined sclerotherapy’s safety, technical efficacy, pain relief, endometrioma recurrence, impact on ovarian reserve, assisted reproductive technology (ART) success, and pregnancy outcomes. Additionally, we assessed how sclerotherapy compares to endometriotic cystectomy.

## 2 Methods

### 2.1 Protocol

The study protocol was designed *a priori* and agreed upon by all authors. We conducted an overview (systematic review) of systematic reviews following the recommendations of Smith et al. (18) and the guidelines of Preferred Reporting Items for Systematic Reviews and Meta-Analyses (PRISMA) (19, 20). The overview was registered in advance on the International Prospective Register of Systematic Reviews (PROSPERO—CRD42024595209).

### 2.2 Eligibility criteria, information sources, search strategy

We conducted an extensive database search on PubMed, Medline (Web of Science platform), Epistemonikos, the Cochrane Library, and PROSPERO. The search in these databases focused on publications in the English literature from their inception until March 31, 2025. The search terms (including their MeSH) used were endometriosis OR endometrioma AND sclerotherapy. The investigation was limited to manuscripts published in peer-reviewed journals and human studies. After the selection process, we screened the bibliographies of eligible manuscripts for other potentially suitable papers. All sclerosing agents were considered for inclusion in the search strategy.

Systematic reviews (with or without meta-analysis) that examined the safety and efficacy of sclerotherapy in women with ovarian endometrioma at reproductive age were applicable for evaluation. Specifically, reviews that explored the procedure efficacy, pain relief, the recurrence rate of endometrioma, the impact on ovarian reserve, the ART outcome, and the pregnancy outcome were eligible. Furthermore, systematic reviews that compared sclerotherapy safety and efficacy to endometriotic cystectomy were qualified.

Narrative reviews and opinion papers were excluded from the evaluation. Systematic reviews targeting only women with non-endometriotic ovarian cysts, gonadal or non-gonadal malignancies, and polycystic ovary syndrome were also excluded. Additionally, systematic reviews focused on females in adolescence, perimenopause, or postmenopause were excluded from the evaluation. Moreover, papers assessing women undergoing other minimally invasive approaches, such as endometrioma ablation using electrocoagulation or plasma energy and laser vaporization, were excluded. Data presented exclusively as abstracts at scientific meetings were discounted. Furthermore, reviews with discernible sampling bias or inappropriate design were excluded.

### 2.3 Study selection

All studies relevant to the research question were retrieved. Two reviewers (JSY and NS) independently screened, reviewed, and selected relevant publications for this overview. The screening process for systematic reviews consisted of two stages, e.g., title/abstract, followed by full-text. We resolved disagreements regarding inclusion, quality assessment, and data extraction through discussion, consensus, or mediation with a third reviewer (HST).

### 2.4 Quality assessment and study overlap degree

Two independent reviewers (JSY and NS) utilized the AMSTAR2 tool to assess the quality of eligible systematic reviews (17). This tool was selected because current meta-analyses often include both randomized and non-randomized intervention studies in their evaluations. AMSTAR2 helps decision-makers identify high-quality systematic reviews that cover both types of studies.

The evaluation of eligible systematic reviews was conducted using the sixteen domains of AMSTAR2. Each domain was categorized as “yes,” “partial yes,” or “no” based on whether it was fully implemented (and documented), partially applied, or not implemented, respectively, in each systematic review. Based on the critical and non-critical domains defined by AMSTAR2, the overall evidence was rated as high, moderate, low, or critically low (17).

We utilized a citation matrix to evaluate the overlap among studies analyzed in the included systematic reviews. This allowed us to calculate the corrected cover area (CCA) according to Pieper et al. (21).

### 2.5 Outcome measures

To properly evaluate the use of sclerotherapy for managing endometriomas, we first looked at whether it was used as a primary treatment (without previous surgery) or as a secondary treatment (after recurrence). We focused on assessing the technical efficacy of the procedure, its impact on pain relief, and the occurrence of any adverse events. Additionally, we studied the likelihood of endometrioma recurrence after the procedure and the effect of sclerotherapy on measures of ovarian reserve. We also examined how sclerotherapy affected pregnancy rates and assisted reproductive technology (ART) outcomes. Furthermore, we compared the outcome measures of sclerotherapy with those of endometriotic cystectomy.

For consistency, outcomes were defined as follows: technical success = successful aspiration and instillation/retention of ethanol without procedural failure; recurrence = reappearance of an endometrioma in the treated ovary on follow-up imaging; pain improvement = reduction or resolution of endometriosis-related pelvic pain as reported by individual studies; pregnancy outcomes = any reported conception, unless specifically defined as spontaneous or ART-related. Where reviews distinguished a clinical pregnancy (ultrasound-confirmed gestational sac) or live birth, this is stated explicitly.

### 2.6 Data extraction and analysis

Two reviewers, JSY and NS, independently performed the data extraction. We created a descriptive table summarizing all systematic reviews on sclerotherapy for managing endometrioma during the reproductive years. For each eligible review, we documented the first author, publication year, the study aims, detailed review objectives, sclerosing agent used, number of studies included in the review, total number of women, number of studies that exclusively used ethanol, the tool used for evaluating the risk of bias in the included studies, and whether a meta-analysis was conducted.

We have created a second descriptive table that inclusively summarizes the results of the outcome measures of all eligible reviews. We collected pooled estimates of the outcome measures from the systematic reviews and meta-analyses and organized them into the table. The remaining results were either calculated or summarized from the data within the systematic reviews. Additionally, we included a comparison of sclerotherapy and endometriotic cystectomy.

### 2.7 Strength of evidence

In systematic reviews that predominantly employed ethanol and performed pooled estimates, we meticulously assessed the strength of evidence for each outcome measure following the GRADE recommendations (22, 23). Two review authors (JSY, NS) independently and thoroughly assessed the certainty of the evidence using GRADE. The level of evidence was evaluated using the five recommended domains of GRADE scoring, which include study limitations (risk of bias), inconsistency, indirectness, imprecision, and publication bias. The overall grading was adversely affected by these factors. To enhance the application of the GRADE scoring tool, we utilized the checklist of questions that was published in 2014 (24).

Since the current systematic reviews include many observational studies, we assessed the possibility of increasing the level of evidence by considering three additional factors in the GRADE system. These factors are a large magnitude of effect, a dose-response gradient, and residual confounders (24). Finally, we classified the levels and certainty of the evidence for each outcome studied on a four-level scale: very low, low, moderate, or high (22, 23).

## 3 Results

### 3.1 Study selection

**Figure 1:**
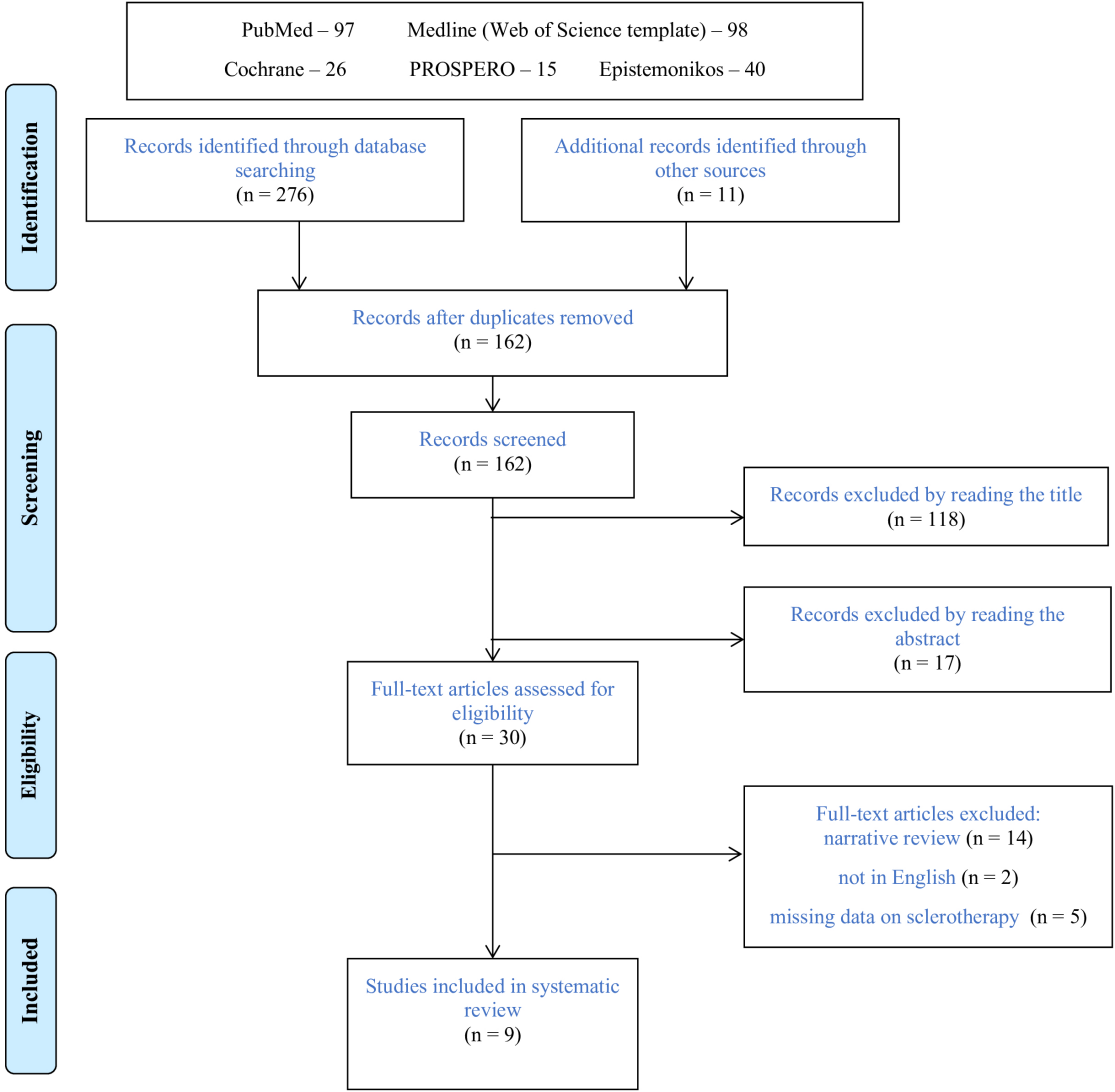
Flow diagram of identified studies.

Database and manual searching identified two hundred seventy-six manuscripts (**Figure 1**). Following exclusions of duplicates and elimination of publications by reading the title and abstract, thirty reviews were qualified for full-text assessment. Among these publications, twenty-one were excluded from the final evaluation; fourteen were narrative reviews (25–38), five did not discuss sclerotherapy (39–43), and two were not in English (44, 45). The remaining nine systematic reviews were eligible for final analysis (14, 15, 46–52).

### 3.2 Study characteristics

As summarized in **Table 1**, nine systematic reviews were eligible for critical appraisal: seven with meta-analysis (14, 15, 47–51) and two without (46, 52). According to the Journal Citation Reports 2023, all of these systematic reviews, except one (15), were published in a Journal with an established impact factor. Six systematic reviews evaluated multiple sclerosing agents, including Ethanol, Methotrexate, Tetracycline, and Lauromacrogol, and three targeted solely Ethanol (15, 49, 52). In five systematic reviews, ethanol was the predominant sclerosing agent (14, 15, 47, 51, 52).

**Table 1:** Summary of systematic reviews exploring sclerotherapy for cases with ovarian endometrioma.

Authors	Journal	Purpose	Objectives studied	Sclerosing agent	Number of studies	Number of studies with ethanol	Total number of women	Risk of bias evaluation	Meta-analysis
Gonçalves et al., 2016 (46)	Int J Gynecol Obstet	Safety and effectiveness of US-guided aspiration and sclerotherapy for repeat endometrioma	Recurrence Pain relief Ovarian reserve Complications Pregnancy rate	Ethanol Methotrexate Tetracycline	8	2	745	no	no
Cohen et al., 2017 (47)	Fertil Steril	Efficacy of sclerotherapy for endometrioma	Recurrence Pain relief Fertility outcome Adverse events	Ethanol Methotrexate Tetracycline	18	13	1,070	Newcastle-Ottowa scale + Cochrane Collaboration's tool	yes
Alborzi et al., 2019 (48)	Reprod Med Biol	Success of 4 different endometrioma treatment approaches	Pregnancy rate	Ethanol Methotrexate	8	1	533 142 Aspiration ± Sclerotherapy	NIH's quality assessment tool	yes
Gao et al., 2022 (49)	Arch Gynecol Obstet	Effect of endometrioma aspiration on IVF/ICSI outcomes	Live birth rate Clinical pregnancy rate Number of oocytes Estradiol peak Gonadotropin dose	Ethanol	10	2	1,207	Newcastle-Ottowa scale + Cochrane risk of bias tool	yes
Garćia-Garćia et al., 2022 (50)	J Minim Invas Gynecol	Recurrence and morbidity after US-guided aspiration with and without sclerotherapy of benign cystic masses	Recurrence Adverse events	Ethanol Lauromacrogol Methotrexate Tetracycline	20	8	1,386 benign cysts 352 endometrioma	Newcastle-Ottowa scale	yes
Kim et al., 2022 (14)	Europ Radiol	Efficacy and safety of US-guided sclerotherapy of endometrioma	Technical success Pain relief Recurrence Ovarian reserve Complications Pregnancy rate Comparison with surgery	Ethanol Lauromacrogol Methotrexate Tetracycline	28	21	1,301	NIH's quality assessment tool	yes
Ronsini et al., 2023 (51)	Medicina	Efficiency of sclerotherapy versus cystectomy for endometrioma	Technical success Recurrence Pregnancy rate Comparison with surgery	Ethanol Methotrexate Tetracycline	29	24	1,642	Newcastle-Ottowa scale	yes
Frankowska et al., 2024 (52)	Int J Mol Sci	Efficacy and safety of US-guided ethanol sclerotherapy for endometrioma	Pain relief Recurrence Complications Ovarian reserve ART outcome Pregnancy outcome	Ethanol	16	16	1,159	Newcastle-Ottowa scale + Cochrane risk of bias 2.0 tool	no
He et al., 2024 (15)	Current Women's Health Review	Efficacy and safety comparison between sclerotherapy and cystectomy for endometrioma	Technical success Pain relief Recurrence Complications Pregnancy rate	Ethanol	6	6	386	Methodological index for non-randomized studies	yes

ART, assisted reproductive technologies; US, ultrasound.

Additionally, all but one systematic review evaluated only women with endometrioma, while another review evaluated other benign pelvic cystic masses, including simple cysts, hydrosalpinx, and other lesions, in addition to endometrioma (50). Furthermore, all systematic reviews evaluated endometrioma aspiration and sclerotherapy; seven compared sclerotherapy with surgery (14, 15, 47–49, 51, 52), and one evaluated other endometrioma management modalities (48).

### 3.3 Quality assessment of systematic reviews

The quality of eligible systematic reviews per the AMSTAR2 tool ranged between moderate to critically low (**Table 2**). The AMSTAR2 sixteen domains were scored as yes, partial yes, or no based on whether it was fully implemented, partially applied, or not implemented, respectively. Following the AMSTAR2 critical and non-critical defined domains (17), the quality appraisal was critically low in three (15, 46, 48), low in four (47, 49, 50, 52), and moderate in two (14, 51) systematic reviews.

**Table 2:** AMSTAR2 quality appraisal of the systematic reviews exploring the impact of US-guided aspiration and sclerotherapy on endometrioma treatment.

	Gonçalves et al., 2016 (46)	Cohen et al., 2017 (47)	Alborzi et al., 2019 (48)	Gao et al., 2022 (49)	Garćia-Garćia et al., 2022 (50)	Kim et al., 2022 (14)	Ronsini et al., 2023 (51)	Frankowska et al., 2024 (52)	He et al., 2024 (15)
1. Research question and inclusion criteria per PICO	PY	PY	Y	Y	Y	PY	Y	Y	Y
2. A priori protocol methods established	N	PY	PY	PY	PY	PY	Y	PY	PY
3. Explanation for the selection of the study design	N	N	N	N	PY	N	PY	N	N
4. A comprehensive literature search strategy	Y	Y	Y	Y	Y	Y	Y	Y	Y
5. Study selection in duplicate	Y	Y	N	Y	Y	Y	Y	Y	Y
6. Data extraction in duplicate	Y	Y	Y	Y	Y	Y	Y	N	Y
7. List of excluded studies with justification	PY	Y	N	PY	PY	Y	PY	PY	PY
8. Included studies in adequate detail	Y	Y	Y	Y	Y	Y	Y	Y	Y
9. Satisfactory technique for assessing the risk of bias	N	Y	Y	Y	Y	Y	Y	Y	PY
10. Sources of funding of included studies reported	N	N	N	N	N	N	N	N	N
11. Appropriate methods for statistical combination of results	NA	Y	Y	Y	Y	Y	Y	NA	PY
12. The potential impact of risk of bias in included studies on statistical estimates	NA	Y	N	N	N	N	N	NA	Y
13. Risk of bias in included studies accounted for in interpretation of results	N	Y	N	Y	Y	Y	Y	N	Y
14. Satisfactory explanation for heterogeneity observed	NA	PY	N	N	PY	PY	N	NA	N
15. Adequate investigation of publication bias	NA	N	N	Y	N	Y	Y	N	N
16. Potential sources of conflict of interest reported	Y	Y	Y	Y	Y	Y	Y	Y	Y
Rating overall confidence per AMSTAR2 critical and non-critical domains	Critically low	Low	Critically low	Low	Low	Moderate	Moderate	Low	Critically low

Y, Yes; PY, partially Yes; N, No; NA, not addressed.

Domains 1, 4, 7, 9, 11, 13, and 15 are the critical domains.

An overlap analysis using a citation index revealed that the nine eligible systematic reviews included 67 studies, with 31 of them analyzed more than once. Using the citation matrix proposed by Pieper et al. (21), the corrected covered area was calculated to be 14.1%. This overlap prevented the conduct of a random-effects meta-analysis for the primary outcomes.

Only seven studies were included in four systematic reviews that compared ethanol sclerotherapy to endometriotic cystectomy and examined pregnancy rates. One study was analyzed four times, while another four were analyzed three times, precluding a random-effects meta-analysis to combine the effect measures for this outcome.

### 3.4 Ethanol sclerotherapy methodology

The practice of ethanol sclerotherapy varied among the studies in each systematic review (**Table 3**). In most cases, aspiration and sclerotherapy were conducted trans-vaginally under ultrasound guidance. In some cases, the trans-abdominal approach was chosen depending on the exact location of the endometrioma. In one of the largest systematic reviews, among the 21 studies that employed ethanol as the sclerosing agent, only six employed the trans-abdominal approach guided by ultrasound or laparoscopy (14).

**Table 3:** Sclerotherapy technique in systematic reviews predominantly employing ethanol.

Authors	Number of studies employing ethanol	Sclerotherapy approach	Needle or catheter	Ethanol concentration	Ethanol volume	Time of sclerotherapy
Cohen et al., 2017 (47)	13	ND	ND	50-100% in 7/13, 95-100%	50-100% of cyst volume and <60-100 ml	5-15 minutes 11 left in situ 4
Kim et al., 2022 (14)	21	US-guided 21	ND	95-100%	20-100% of cyst volume	5-30 minutes 13 left in situ 10
Ronsini et al., 2023 (51)	24	US-guided 23 LPS-guided 1	Needle 21 Catheter 3	20-100% in 17/24, 95-100%	25-100% of cyst volume and <60-100 ml	1 - 20 minutes 15 left in situ 4 ND 5
Frankowska et al., 2024 (52)	16	US-guided 16	ND	20-100% in 11/16, 95-100%	60-90% of cyst volume	5-20 minutes 10 left in situ 4 ND 2
He et al., 2024 (15)	6	US-guided 6	Needle 5 Catheter 1	ND	25-80%of cyst volume and <60-100 ml	5-20 minutes 5 left in situ 1

US, ultrasound, LPS – laparoscopy, ND – not disclosed.

The procedure was typically carried out under sterile conditions. The endometrioma was usually punctured and aspirated using a needle ranging from 17 to 20 gauge and 20 to 25 cm long. In some cases, an 8.5-F pigtail catheter was utilized instead. Generally, the endometrioma was aspirated to remove its contents and then flushed with a saline solution before introducing ethanol.

The concentration of ethanol used in the studies ranged from 20% to 100%, with most studies employing 95% to 100% dosing (about two-thirds of studies). The volume of ethanol injected depended on the endometrioma size and varied between 20% and 100% of the endometrioma volume. In numerous studies, the maximal volume of ethanol used was below 60–100 ml to prevent spillage and rupture.

The duration of ethanol sclerotherapy varied across different studies. Some studies utilized irrigation, while others retained the ethanol, with approximately two-thirds of cases involving irrigation and one-third retaining the ethanol. In the irrigation cases, the duration of ethanol contact varied widely, ranging from one to 30 minutes. In most instances, the duration fell between 5 and 20 minutes.

### 3.5 Synthesis of results of systematic reviews

Nine eligible reviews focused on various safety and efficacy aspects of sclerotherapy for endometrioma management in women of reproductive age. The results are summarized in **Table 4**. Seven systematic reviews also compared sclerotherapy with the surgical approach, specifically endometriotic cystectomy. The main findings are focused on accumulated evidence of these aspects, specifically when employing ethanol as the sclerosing agent and when data was pooled for quantitative estimates.

**Table 4:** The effect of sclerotherapy on endometrioma management, exploring safety and efficacy aspects.

	Sclerotherapy as primary or secondary treatment	Technical efficacy	Pain relief	Endometrioma recurrence	Impact on ovarian reserve	Adverse events	Pregnancy rates and IVF results	Comparison with endometriotic cystectomy
Gonçalves et al., 2016 (46)	secondary	efficient	yes	sclerotherapy is superior to aspiration alone	AFC increase	ND	not conclusive	NE
Cohen et al., 2017 (47)	combined	NE	yes	ethanol retention is better than irrigation	AFC increase	ND	compared to no treatment, similar number of oocytes and clinical pregnancy rate	more oocytes, similar clinical pregnancy rate
Alborzi et al., 2019 (48)	combined	NE	NE	NE	NE	NE	NE	similar fertilization rate and clinical pregnancy rate
Gao et al., 2022 (49)	ND	NE	NE	NE	NE	NE	compared to no treatment, similar number of oocytes and clinical pregnancy rate	more oocytes, similar clinical pregnancy rate
Garćia-Garćia et al., 2022 (50)	combined	NE	NE	sclerotherapy is better than aspiration alone 14% vs 52.3%	NE	minor ND major 3/302 (1%)	NE	NE
Kim et al., 2022 (14)	combined	pooled rate 98.3%	pooled rate 85.6%	pooled rate 13.8% sclerotherapy time ≤ 10 min. 20.9% > 10 min. 11.2% (NS)	no change in AMH	pooled rate minor 11.0% major 1.7%	37.3% pooled pregnancy rate ND if spontaneous or IVF, sclerotherapy time has no effect	lower recurrence and better clinical pregnancy rate with sclerotherapy
Ronsini et al., 2023 (51)	ND	95.0% (844/888)	NE	20.3% (218/1,387)	NE	NE	pregnancy rate 33.2% (375/1,128) ND if spontaneous or IVF	comparable recurrence rate and pregnancy rate
Frankowska et al., 2024 (52)	combined	NE	yes, but data not conclusive	range 0-62.5%, not conclusive, depends on installation time	AMH no change, AFC increase	minor low < 12% major very low	comparable number of oocytes, number of embryos, fertilization rate, implantation rate, and pregnancy rate, but data not conclusive	lower AMH after surgery, comparable recurrence rate
He et al., 2024 (15)	ND	100% (53/53)	51% (57/111)	16.7% (31/186)	NE	total 17.7% (23/130)	Pregnancy rate 40.2% (49/122) ND if spontaneous or IVF	comparable technical efficacy, recurrence, adverse events, and pregnancy rates

ND, not disclosed; NE, not examined; figures between brackets are calculated from data within the systematic reviews.

It is essential to note that all eligible systematic reviews included combined studies that evaluated women with endometrioma undergoing sclerotherapy as a primary (intact endometrioma) and secondary (endometrioma recurrence) treatment option.

#### 3.5.1 Adverse events

Adverse events secondary to sclerotherapy were evaluated in five systematic reviews (14, 15, 47, 50, 52). In four of them, ethanol was the leading sclerosing agent (14, 15, 47, 52). Combined, the adverse events were defined as minor, including abdominal or pelvic pain, postoperative fever, vasovagal syncope, mild edema of the lower limbs, vaginal bleeding, pelvic inflammation, gastrointestinal reaction, ethanol leakage to the peritoneal cavity, and incomplete cyst aspiration. Major complications included alcohol intoxication and abscess formation (14, 47, 52). The minor complication rate ranged from 0.0% to 36.8% across different studies, with a pooled estimate of 11.0% (95% CI, 7.1–16.5%; *I^2^* = 74%) (14). The major complications rate ranged between 0.0% and 7.1%, with a pooled estimate of 1.7% (95% CI, 1.0–2.8%; *I^2^* = 0%) (14). The rate of minor complications was significantly lower when exposure to ethanol was less than 10 minutes compared to more than 10 minutes, while major complications did not differ.

#### 3.5.2 Technical efficacy

Three systematic reviews, employing predominantly ethanol, evaluated the technical efficacy of sclerotherapy and reported a high success rate, ranging from 95% to 100% (14, 15, 51). Notably, the review that reported 100% success summarized only 53 cases (15), whereas the two others summarized a large cohort of women undergoing endometrioma sclerotherapy (n = 650-888), with a technical efficacy of 95-98.3% (14, 51). Kim et al. reported that the pooled technical success rate of sclerotherapy was 98.3% (95% CI, 96.8–99.1%; *I^2^* = 0%) (14).

#### 3.5.3 Pain relief

Four systematic reviews assessing predominantly ethanol sclerotherapy evaluated pain relief (14, 15, 47, 52). Overall, sclerotherapy improved endometriosis-associated pain; however, the success rate was not uniformly reported across the reviews. Only one review performed a quantitative estimate on this topic, finding a pooled pain resolution or marked improvement in 85.9% (95% CI, 73.9–92.9%; *I^2^* = 48%) of cases (n = 150) (14).

#### 3.5.4 Endometrioma recurrence

Seven systematic reviews evaluated endometrioma recurrence following sclerotherapy (14, 15, 46, 47, 50–52). Five employed predominantly ethanol (14, 15, 47, 51, 52). These reviews showed that ethanol retention in endometrioma had better outcomes than irrigation, particularly with installation time exceeding 10 minutes (14, 47). After a follow-up of at least six months, the recurrence rate varied widely among the studies between 0.0 and 62%, with a pooled estimate of 13.8% (95% CI, 9.4–19.9%; *I^2^* = 75%) (n = 1,121) (14). Direct comparison within the same setting showed that the odds ratio of endometrioma recurrence rate between sclerotherapy of > 10 min and ≤ 10 min was 0.2 (95% CI, 0.1 – 0.8; *I^2^* = 54%), favoring extended exposure (n = 357) (14). Likewise, the risk of endometrioma recurrence within 12 months was 3.47 times higher in the ethanol irrigation group compared to the retention group (95% CI 1.85–6.51, *I^2^* = 25%) (n = 319) (47).

#### 3.5.5 Impact on ovarian reserve

The impact of sclerotherapy on ovarian reserve was assessed in three systematic reviews, which primarily employed ethanol (14, 47, 52). Two ovarian reserve measures, antral follicle count (AFC) and anti-Müllerian hormone (AMH), were utilized for this assessment. Two systematic reviews have shown increased AFC following sclerotherapy (47, 52). Conversely, two others did not find a change in serum AMH levels (14, 52). Only one systematic review with a small number of pooled women (n = 60) analyzed this topic quantitatively (14). The pooled mean difference in serum AMH before and after sclerotherapy was -0.01 ng/mL (95% CI, -0.04 − 0.03; *I^2^* = 0%), demonstrating no significant change in ovarian reserve. The impact of ethanol sclerosing time on ovarian reserve was not examined in any systematic review. Notably, the effect of sclerotherapy on ovarian reserve measures over time was not assessed in any of the eligible reviews.

#### 3.5.6 Pregnancy rate

Pregnancy outcomes following sclerotherapy are reported inconsistently. Some reviews included spontaneous conceptions, others ART-related pregnancies (IVF/ICSI), and many did not distinguish between them. In our synthesis, “pregnancy” refers to any reported conception unless explicitly stated as “spontaneous pregnancy” or “clinical pregnancy” following ART.

Specifically, several systematic reviews addressed the pregnancy rate following sclerotherapy (14, 15, 46, 47, 49, 51, 52). In five of them, ethanol was the primary sclerosing agent (14, 15, 47, 51, 52). Most of these publications did not disclose whether the pregnancy was achieved spontaneously or after IVF (14, 15, 51, 52). Furthermore, most reviews did not clearly define pregnancy (14, 51, 52). This is clarified in **Table 4**, under ‘Pregnancy Rates and IVF Results’.

Only one review performed a pooled estimate of pregnancy rate, calculated as 37.6% (95% CI, 30.2–45.6%; *I^2^* = 64%) (n = 538) (14). Notably, in this study, subgroup analysis revealed no difference in pregnancy rates between sclerotherapy ≤ 10 and > 10 minutes, respectively.

#### 3.5.7 Comparison with the surgical approach

Seven systematic reviews compared sclerotherapy to endometriotic cystectomy (14, 15, 47–49, 51, 52). Five employed ethanol as the predominant sclerosing agent (14, 15, 47, 51, 52), and four performed quantitative data analyses (**Table 4**). These reviews evaluated one or more outcome measures, including the technical efficacy, recurrence rate, adverse events, the number of oocytes in IVF, and pregnancy rates.

In the review by Cohen et al, the authors pooled and analyzed three studies involving 178 women and found that sclerotherapy resulted in a significantly higher number of oocytes at IVF retrieval than endometriotic cystectomy. The mean difference was 2.7 (95% CI 0.98–4.4; *I^2^* = 71%) (47). However, there was no significant difference in clinical pregnancy rates (n = 163), with an odds ratio of 1.63 (0.91–2.9; *I^2^* = 28%). It is worth noting that when comparing sclerotherapy to no treatment, the number of retrieved oocytes and clinical pregnancy rates were similar, pooling three studies, including 164 women.

Similarly, Ronsini et al. analyzed seven comparative studies involving 370 women and showed a non-significant difference in pregnancy rate favoring sclerotherapy over endometriotic cystectomy, with an odds ratio of 0.47 (95% CI 0.21–1.09; *I^2^* = 60%) (51). In the same systematic review evaluating endometrioma recurrence, four studies that included 303 women showed comparable results between the two management modalities, with an odds ratio of 0.87 (95% CI 0.18–4.32; *I^2^* = 62%).

Likewise, He et al. analyzed six comparative studies, including 386 women. The endometrioma recurrence rate did not significantly differ between ethanol sclerotherapy and endometriotic cystectomy, with an OR of 1.57 (95% CI 0.39 – - 6.25; *I^2^* = 54%) (15). Of note, when sclerotherapy time was <10 minutes, sub-group analysis revealed a significantly higher risk of recurrence (n = 91) with an OR of 22.01 (95% CI 3.31 – -146.26; *I^2^* = 0%). Conversely, comparable results were achieved between the groups when the irrigation time was > 10 minutes. Comparison of adverse events (n = 285) was also comparable between the two groups with an OR of 1.03 (95% CI 0.20 – -5.38; *I^2^* = 43%). Furthermore, the clinical pregnancy rate did not differ significantly between the groups (n = 235) with an OR of 1.67 (95% CI, 0.74 –3.75; *I^2^* = 34%). Subgroup analysis revealed that the duration of ethanol irrigation, whether lower or higher than 10 minutes, did not impact clinical pregnancy rates.

Conversely, Kim et al. pooled and analyzed three studies involving 189 women and found that sclerotherapy significantly increased the pregnancy rate in comparison to endometriotic cystectomy, with an odds ratio of 2.0 (95% CI, 1.0–3.8; *I^2^* = 0%) (14).

### 3.6 GRADE strength (certainty) of evidence

**Table 5** summarizes the strength of evidence for the main outcome measures in eligible systematic reviews primarily focusing on ethanol sclerotherapy that attained quantitative estimates. The strength of evidence of these outcome measures was evaluated using the GRADE tool. The eligible systematic reviews included both experimental (RCT) and observational studies. As most cases involved observational studies, the evidence initially in our overview started with low quality following the GRADE approach (23). Furthermore, a serious limitation was present in all eligible systematic reviews, specifically in the ‘limitations’ domain (risk of bias). Therefore, since serious limitations preclude upgrading the level of evidence according to the GRADE recommendations (23), we decided not to consider the magnitude of effect, dose-response gradients, and residual confounders to promote the primary evaluation’s results.

**Table 5:** GRADE strength of evidence score for significant outcomes reported in the overview of systematic reviews assessing ethanol sclerotherapy for endometrioma management in the reproductive age*.

Outcome	Systematic review	Intervention	N (n)	Effects (95% CI) and heterogeneity	GRADE assessment					Strength of evidence
					Limitations (Risk of bias)	Inconsistency	Indirectness	Imprecision	Publication bias	
Technical efficacy	Kim et al., 2022 (14)	Sclerotherapy	15 (650)	Pooled estimate 98.3% (96.8 - 99.1%) *I^2^* 0%	serious	no	no	no	no	Low
Pain relief	Kim et al., 2022 (14)	Sclerotherapy	8 (150)	Pooled proportion 85.9% (73.9 – 92.9%) *I^2^* 48%	serious	yes	yes	yes	no	Very low
Endometrioma recurrence	Cohen et al., 2017 (47) Kim et al., 2022 (14)	Irrigation versus retention Sclerotherapy Sclerotherapy > 10 versus < 10 minutes	3 (319) 23 (1,002) 4 (357)	OR 3.47 (1.85 - 6.51) *I^2^* 54% Pooled estimate 13.8% (9.4 – 19.9%) *I^2^* 75% OR 0.2 (0.1 - 0.8) *I^2^* 54%	serious serious serious	no yes yes	no yes no	yes yes no	no no no	Low Very low Low
Adverse events	Kim et al., 2022 (14)	Sclerotherapy	25 (1,117)	Pooled estimate Minor 11.0% (7.1 – 16.5%) *I^2^* 74% Major 1.7% (1.0 – 2.8%) *I^2^* 0%	serious	no	yes	no	no	Low
Impact on ovarian reserve	Kim et al., 2022 (14)	Sclerotherapy impact on AMH levels	4 (130)	Pooled standard mean difference -0.01 ng/mL (-0.04 – 0.03) *I^2^* 0%	serious	no	yes	yes	yes	Very low
Pregnancy rates	Kim et al., 2022 (14)	Sclerotherapy	19 (538)	Pooled estimate 37.6% (30.2 – 45.6%) *I^2^* 46%	serious	yes	yes	yes	no	Very low
IVF results	Cohen et al., 2017 (47)	Number of oocytes sclerotherapy versus no treatment	3 (148)	-0.51 (-2.23 – 1.21) *I^2^* 17%	serious	no	yes	yes	no	Very low
Comparison of sclerotherapy with endometriotic cystectomy	Cohen et al., 2017 (47)	Number of oocytes	2 (178)	2.71 (0.98 – 4.44) *I^2^* 71%	serious	no	yes	yes	no	Very low
		Clinical IVF pregnancy	3 (214)	OR 1.63 (0.91 – 2.90) *I^2^* 28%	serious	no	yes	yes	no	Very low
	Kim et al., 2022 (14)	Pregnancy rate	4 (189)	OR 2.0 (1.00 – 3.80) *I^2^* 0%	serious	yes	yes	yes	no	Very low
	Ronsini et al., 2023 (51)	Recurrence rate	4 (303)	OR 0.87 (0.18 – 4.32) *I^2^* 62%	serious	yes	yes	no	no	Low
		Pregnancy rate	7 (370)	OR 0.47 (0.21 – 1.09) *I^2^* 70%	yes	yes	yes	no	no	Low
	He et al., 2024 (15)	Adverse events	5 (285)	OR 1.03 (0.20 – 5.38) *I^2^* 34%	serious	yes	yes	yes	no	Very low
		Recurrence rate	5 (360)	OR 1.57 (0.39 – 6.25) *I^2^* 57%	serious	yes	yes	no	no	Very low
		Pregnancy rate	5 (235)	OR 1.67 (0.74 – 3.75) *I^2^* 34%	serious	no	no	yes	no	Low

*Reasons for downgrading: “Limitations” reflects methodological weaknesses (mainly observational studies, retrospective designs, risk of bias); “Inconsistency” refers to heterogeneity of effect estimates or outcome definitions; “Indirectness” refers to use of mixed agents, varied outcome definitions, or lack of direct evidence on ethanol; “Imprecision” reflects wide confidence intervals or small pooled samples; “Publication bias” reflects likelihood of selective reporting of positive outcomes.

The strength of evidence regarding the technical efficacy and adverse events of ethanol sclerotherapy in managing endometrioma was low. The pooled estimate of technical efficacy was 98.3%, and the minor and major adverse events were 11.0% and 1.7%, respectively (14). Despite the low strength of evidence, the large number of women involved in the evaluation (650 and 1,117) and the short-term effect on these outcomes increase the evidence, may suggest that this minimally invasive approach could be a feasible option for treating endometrioma.

Moreover, there was a low level of evidence to show that ethanol retention is superior to irrigation, and ethanol exposure of > 10 minutes is more efficient than ≤ 10 minutes in preventing endometrioma recurrence (14, 47).

Conversely, sclerotherapy’s impact on pain relief, ovarian reserve, pregnancy rates, and IVF results had a very low level of evidence, suggesting that the estimated effects are very uncertain.

Four systematic reviews have compared ethanol sclerotherapy to endometriotic cystectomy, focusing on adverse events, recurrence rate, pregnancy rates, number of oocytes, and clinical pregnancy rates in the context of IVF (14, 15, 47, 51). The strength of evidence varied from low to very low and, at times, was controversial, making it difficult to draw definitive conclusions. Of note, two systematic reviews reported similar pregnancy rates between sclerotherapy and endometriotic cystectomy (n = 370 and 235, respectively) (15, 51), whereas a third review showed an advantage for sclerotherapy (n = 189) (14). However, the level of evidence was low in the first two reviews and very low in the third, precluding a definitive conclusion.

## 4 Discussion

### 4.1 Main findings

Our comprehensive critical overview of systematic reviews indicates that sclerotherapy, specifically with ethanol, is a feasible and readily applicable management modality for women with endometrioma during their reproductive years. This finding supports its application as a minimally invasive management approach for women facing infertility or those who have not yet achieved their reproductive goals. Sclerotherapy appears to preserve ovarian reserve better than ovarian cystectomy.

Although each primary outcome measure had only one estimate among the nine systematic reviews (which precluded the pooling of effect measures), our overview shows that sclerotherapy has a high technical efficacy of 95.0-98.3%. Additionally, the rates of adverse events were low, with minor events reported at 11.0-12.0% and major adverse events at 1.0-1.7%. Although the strength of the evidence was assessed as low, the analysis involved a large number of women, and the effect was observed over a short time, supporting the feasibility of this minimally invasive approach for managing endometrioma in women of reproductive age.

Furthermore, there is low-strength evidence that ethanol retention is more effective than irrigation, and sclerotherapy for more than 10 minutes is better than less than 10 minutes in preventing endometrioma recurrence.

The impact of ethanol sclerotherapy on pain relief, ovarian reserve, pregnancy rate, and IVF results are based on very low evidence strength, suggesting uncertainty and precluding definitive conclusions. In addition, systematic reviews that compared sclerotherapy to endometriotic cystectomy, although showing comparable pregnancy rates, had low to very low evidence strength and sometimes provided controversial estimates, making reliable conclusions at this stage challenging to reach.

### 4.2 Comparison with existing literature

Sclerotherapy is the controlled, therapeutic use of sclerosants to destroy undesired target tissues. Ethanol sclerotherapy was first applied in 1980 and was used to manage endometrioma in 1988 (53, 54). Since then, it has gained popularity worldwide. Its mechanism of action involves cytotoxic damage induced by the denaturation and extraction of surface proteins, hypertonic dehydration of cells, and coagulation and thrombosis in the presence of blood products, leading to fibrinoid necrosis (55). Dosing should not exceed 1 mL/kg because this dose can lead to systemic blood alcohol concentrations of up to 0.07%, posing the risk of alcohol intoxication. The risk of alcohol intoxication in our overview, under these regulations, was negligible. Furthermore, the rates of other minor and major adverse events were reasonable in our overview, supporting the feasibility of this management modality for endometrioma.

Extensive literature continuously discusses whether endometrioma could reduce ovarian reserve by itself. Evidence exists on both sides, but the proof seems inconclusive (56, 57). A recent systematic review of prospective studies (n = 1,272) found that intact bilateral endometrioma, compared to unilateral endometrioma, a surrogate measure of more advanced disease, did not significantly affect preoperative serum AMH levels, challenging that endometrioma per se reduces functional ovarian reserve (6). Conversely, there is convincing evidence that ovarian cystectomy significantly harms ovarian reserve in a non-reversible way (5, 6), and may lead to ovarian insufficiency and early menopause (11–13). Our overview implies that ethanol sclerotherapy did not impair ovarian reserve; however, the strength of evidence was low. Additionally, future research on this matter may be beneficial in refining ethanol dosage, volume, and sclerosing duration to optimize its effect on ovarian reserve and endometrioma recurrence.

Endometrioma recurrence after endometriotic cystectomy is also a primary concern. Based on short-term and long-term follow-up studies, the reported recurrence rate after endometriotic cystectomy is high, estimated at 21.5% at two years and 40-50% at five years (58, 59). Furthermore, there is a high rate of repeat surgeries (60, 61). In a recent large-scale, population-based, long-term study on women who underwent surgery for endometriosis, it was found that 1 in 4 individuals following minor surgery and 1 in 5 following major conservative surgery (with ovarian preservation) required additional endometriosis surgery (61).

In our overview, the recurrence rate varied considerably, ranging from 16% to 67%. However, our overview of eligible systematic reviews of ethanol sclerotherapy was assessed in combined primary and secondary treatment cases based on short-term follow-up. Future studies should focus on sclerotherapy as a primary management tool with long-term follow-up. In this regard, identifying biomarkers for the diagnosis or recurrence of endometriosis would help track disease progression (62, 63).

In the IVF setting, various systematic reviews have shown that an intact endometrioma reduces the number of retrieved oocytes compared to normal ovaries, but clinical and live birth rates are comparable (64–66). Furthermore, several systematic reviews have shown that endometriotic cystectomy, compared to expectant management, does not improve clinical pregnancy and live birth rates (64, 67–71).

In our overview, we found that using ethanol sclerotherapy to treat endometriomas did not show significant differences in pregnancy rates when compared to expectant management or endometriotic cystectomy. However, it was unclear whether the pregnancies occurred naturally or as a result of IVF treatment. Additionally, the evidence supporting these findings was of low to very low quality. Future studies should investigate whether sclerotherapy offers any advantages over expectant management or endometriotic cystectomy in terms of pregnancy and live birth rates.

Our overview of systematic reviews did not assess the cost-effectiveness of ethanol sclerotherapy. In recent small-scale studies (n = 33-71), ethanol sclerotherapy, compared to endometriotic cystectomy, avoided or reduced hospitalization stays, decreasing health costs substantially (72, 73). One study performed the procedure on outpatients without anesthesia, sedation, or prophylactic antibiotics, reducing health costs eightfold (73). Future studies should pursue ethanol sclerotherapy costs, achieving the best outcome measures at the lowest possible cost.

Ethanol sclerotherapy has its limitations. It cannot be performed when an endometrioma ruptures during the procedure or when there is a risk of malignancy. However, the occurrence of endometrioma rupture and the risk of malignant transformation are both uncommon and sporadic. Among women with gonadal endometriosis-associated ovarian cancer, only 0.028% are under the age of 40, with a calculated risk of 3 to 1000 (74). Utilizing magnetic resonance imaging (MRI) for atypical endometriomas diagnosed by trans-vaginal ultrasound prior to sclerotherapy may aid in distinguishing between benign endometriomas and malignant transformations (75). Furthermore, when other subtypes of endometriosis, such as superficial, deep infiltrating, or adenomyosis, are present, a comprehensive multidisciplinary evaluation is recommended to determine optimal management and treatment planning.

### 4.3 Strengths and limitations

Our overview of the systematic reviews has numerous strengths. It is the first contemporary overview that rigorously evaluates systematic reviews focusing on sclerotherapy, particularly with ethanol, as a minimally invasive treatment for women of reproductive age with ovarian endometriosis. We developed a study protocol that all the review authors agreed upon and registered on PROSPERO beforehand.

The safety and efficacy of endometrioma sclerotherapy management were evaluated by examining various outcome measures. These measures included technical efficacy, pain relief, endometrioma recurrence, impact on ovarian reserve, adverse events, pregnancy rate, and IVF results. Additionally, comparisons with endometriotic cystectomy were also analyzed.

We used five search engines to systematically maximize our results by searching for eligible papers and looking for published and ongoing reviews. Previous publications were narrative and only briefly précised the procedure without delving into the various aspects of sclerotherapy’s safety and effectiveness. Additionally, they did not include details of the search methodology.

We applied the AMSTAR2 tool to evaluate the quality of eligible systematic reviews, incorporating randomized and non-randomized intervention studies. Additionally, we rated each systematic review’s overall evidence according to the critical and non-critical domains defined by AMSTA2 guidelines.

The strength of evidence for each outcome measure in all eligible systematic reviews was evaluated using the GRADE scoring system. Furthermore, the levels and certainty of the evidence for each outcome studied were categorized on a four-level scale: very low, low, moderate, or high, as recommended (22, 23).

In contrast, the overview of systematic reviews we conducted has several limitations. All eligible systematic reviews included an assessment of sclerotherapy as a combined primary and secondary treatment for endometrioma. The reviews pooled cases of endometrioma recurrence after endometriotic cystectomy and others who received sclerotherapy as their initial treatment. This made it challenging to genuinely assess sclerotherapy as a primary management modality in women with endometrioma.

Additionally, the inclusion of both investigational randomized and observational non-randomized (largely retrospective) studies of intervention influenced the quality of systematic reviews. This was assessed using the MASTAR2 tool, which ranged from moderate to critically low. Moreover, it influenced the level of evidence of the outcome measures, as assessed by the GRADE tool, and the strength of recommendations.

As demonstrated in **Table 5**, the primary outcome measures in the included systematic reviews had only one estimate each. Thus, a random-effects meta-analysis was not performed to pool together effect measures for these outcomes. Furthermore, while four systematic reviews comparing ethanol sclerotherapy to endometriotic cystectomy had more than three estimates for two of the primary outcome measures (endometrioma recurrence and pregnancy rates), the overlap between the included studies was high, which precluded pooling the effect measures.

Also, endometrioma laterality (uni- or bilateral) and diameter were not considered when assessing sclerotherapy safety and efficacy. In cases following endometriotic cystectomy, laterality significantly influences the decline in ovarian reserve measures following surgery (6). Furthermore, endometrioma size may impact ovarian reserve since it has been suggested to affect ovarian responsiveness during IVF treatment (76). These endometrioma characteristics are essential when assessing the efficacy and safety of sclerotherapy and should be considered in future research.

Furthermore, the available systematic reviews did not adequately address the impact of sclerotherapy on ovarian reserve. While some reviews touched on this topic using AMH or AFC, none did so proficiently. The rationale for employing sclerotherapy, particularly with ethanol, aimed to avoid the significant decline of ovarian reserve caused by endometriotic cystectomy. Notably, none of the eligible systematic reviews assessed ovarian reserve measures following sclerotherapy over time. Additionally, it is essential to note that AMH is a more sensitive measure of ovarian reserve in this setting, and AFC is not recommended in women with endometrioma (77).

## 5 Conclusions

Ethanol sclerotherapy is a feasible, minimally invasive option for managing endometriomas during reproductive age. The procedure’s high technical efficacy, broad availability, and low incidence of adverse events provide sufficient evidence supporting its use as an alternative to endometriotic cystectomy. Further research should establish ethanol sclerotherapy’s effects on pain relief, ovarian reserve, endometrioma recurrence, and clinical pregnancy rates.

Based on the evidence synthesized in this umbrella review of ethanol sclerotherapy for endometrioma management, we suggest practical considerations for clinical application, summarized in **Table 6**.

**Table 6:** Practical considerations for ethanol sclerotherapy in women with ovarian endometrioma.

Patient selection
◦ Women of reproductive age with infertility, planning a future pregnancy, or experiencing symptomatic or recurrent endometriomas, especially when preserving ovarian reserve is important.
◦ Exclude cases with suspected malignancy; MRI is recommended for atypical ultrasonographic findings.
◦ Can be applied in both primary and recurrent settings, but individualized decision-making is essential.

Contraindications
◦ Suspicion or confirmation of malignancy.
◦ Cyst rupture or challenges in safely accessing the endometrioma.
◦ Allergy or contraindication for ethanol use.

Procedural parameters
◦ Ethanol concentration: most studies used 95–100%.
◦ Maximum dose: ≤1 mL/kg body weight to minimize systemic toxicity.
◦ Injected volume: typically 20–100% of aspirated cyst fluid, not exceeding 60– 100 mL.
◦ Technique: transvaginal ultrasound guidance is standard; trans-abdominal or laparoscopic guidance in selected cases.
◦ Retention vs irrigation: retention is preferred over irrigation.
◦ Instillation time: >10 minutes is associated with lower recurrence compared to ≤10 minutes.

Expected outcomes
◦ High technical success (95–98%).
◦ Low complication rates (major complications 1–2%).
◦ Recurrence reduced with ethanol retention and longer instillation times.
◦ Potential advantage in preserving ovarian reserve compared with cystectomy, though the certainty of evidence remains low to very low.

Practical considerations
◦ Requires careful monitoring because of limited long-term evidence on ovarian reserve, pain, and live birth rates.
◦ Should be performed in centers with expertise in reproductive imaging and interventional gynecology/radiology.
◦ Multidisciplinary counseling is advisable for women seeking fertility treatment.

Future research should prioritize evaluating sclerotherapy as a primary management strategy for endometriomas, in comparison with expectant management and cystectomy. Particular emphasis should be placed on its longitudinal impact on ovarian reserve, using serum AMH as a biomarker, as well as on endometrioma recurrence, clinical pregnancy, and live birth rates. Both spontaneous and IVF-conceived pregnancies warrant assessment. The influence of ethanol exposure duration during sclerotherapy on ovarian reserve should be systematically investigated. Furthermore, endometrioma diameter and laterality should be incorporated as variables in such evaluations, and cost-effectiveness analyses should complement clinical outcomes to establish the overall value of this approach.

## Data Availability

The original contributions presented in the study are included in the article/**Supplementary Material**. Further inquiries can be directed to the corresponding author.
