# Supplementary material for: Ethanol sclerotherapy for management of endometriomas: an overview of systematic reviews
Source: Front Endocrinol (Lausanne). 2025 Nov 18;16:1612899. doi: 10.3389/fendo.2025.1612899 (PMC12670379; doi:10.3389/fendo.2025.1612899)
Supplement: Supplementary file 1 [file DataSheet1.docx]

|  |  | **Systematic reviews** | | | | | | | | |
| --- | --- | --- | --- | --- | --- | --- | --- | --- | --- | --- |
|  | Primary  studies | HE 2024 | FRANKOWSKA 2024 | RONSINI  2023 | GAO 2022 | KIM 2022 | GARCIA-GARCIA 2021 | ALBORZI 2019 | COHEN 2017 | GONCALVES  2016 |
| 1 | Ron El 1991 |  |  |  |  |  | X |  |  |  |
| 2 | Bret 1992 |  |  |  |  |  | X |  |  |  |
| 3 | Giorlandino 1993 |  |  |  |  |  | X |  |  |  |
| 4 | Weinraub 1994 |  |  |  |  |  | X |  |  |  |
| 5 | Chang 1997 |  |  | X |  | X | X |  | X | x |
| 6 | Mesogitis 2000 |  |  | X |  |  |  |  | X |  |
| 7 | Noma 2001 | X | X | X |  | X |  |  | X |  |
| 8 | Koike 2002 |  | X | X |  | X |  |  | X |  |
| 9 | Petrovic 2002 |  |  |  |  |  | X |  |  |  |
| 10 | Suganuma 2002 |  |  | X | X |  |  | X | X |  |
| 11 | Acien 2003 |  |  |  |  |  |  |  |  | X |
| 12 | Chan 2003 |  |  |  |  |  |  |  |  | X |
| 13 | Messalli 2003 |  |  |  |  | X |  |  |  |  |
| 14 | Alborzi 2004 |  |  |  |  |  |  | X |  |  |
| 15 | Fisch 2004 |  |  | X |  | X | X | X | X |  |
| 16 | Pabuccu 2004 |  |  |  | X |  |  | X |  |  |
| 17 | Mesogitis 2005 |  |  |  |  | X |  |  |  |  |
| 18 | Busacca 2006 |  |  |  |  |  |  | X |  |  |
| 19 | Demirol 2006 |  |  |  |  |  |  | X |  |  |
| 20 | Ikuta 2006 |  | X | X |  | X | X |  | X |  |
| 21 | Qublan 2006 |  |  |  | X |  |  |  |  |  |
| 22 | Agostini 2007 |  |  | X |  | X |  |  | X | X |
| 23 | Alborzi 2007 |  |  |  |  |  |  | X |  |  |
| 24 | Hammadieh 2008 |  |  |  |  |  | X |  |  |  |
| 25 | Hsieh 2009 |  | X | X |  | X |  |  | X | X |
| 26 | Yazbech 2009 | X | X | X | X | X | X |  | X |  |
| 27 | Firouzabadi 2010 |  |  |  | X |  |  |  |  |  |
| 28 | Gatta 2010 |  |  | X |  | X |  |  | X |  |
| 29 | Jiang 2010 |  |  |  |  |  | X |  |  |  |
| 30 | Kukura 2010 |  |  |  |  |  | X |  |  |  |
| 31 | Andre 2011 |  | X | X |  | X |  |  | X |  |
| 32 | Kafali 2011 |  |  |  |  | X |  |  |  |  |
| 33 | Shawki 2011 |  |  | X |  | X |  |  | X |  |
| 34 | Wang 2011 |  |  | X |  | X |  |  | X |  |
| 35 | Zhu 2011 |  |  |  |  |  |  |  |  | X |
| 36 | Guo 2012 |  |  |  | X |  |  |  |  | X |
| 37 | Kars 2012 |  |  |  |  |  | X |  |  |  |
| 38 | Shawki 2012 |  |  |  |  | X |  |  |  |  |
| 39 | Aflatoonian 2013 |  | X | X |  |  | X |  | X |  |
| 40 | Chang 2013 |  |  |  |  |  |  |  |  | X |
| 41 | Lee 2014 |  | X | X | X | X |  |  | X |  |
| 42 | Nikolaou 2014 |  |  |  |  |  | X |  |  |  |
| 43 | Begum 2015 |  | X | X |  | X |  |  |  |  |
| 44 | Garcia-tejedor 2015 |  |  | X |  | X | X |  | X |  |
| 45 | Wang 2015 |  |  | X |  | X |  |  | X |  |
| 46 | Wu 2015 |  |  |  |  | X | X |  |  |  |
| 47 | Castellarnau 2016 |  |  |  |  |  | X |  |  |  |
| 48 | Zhang 2016 |  |  |  | X |  |  |  |  |  |
| 49 | Li 2017 |  |  |  | X |  |  |  |  |  |
| 50 | Alborzi 2018 |  |  |  |  | X |  |  |  |  |
| 51 | Bila 2019 |  |  |  |  |  |  | X |  |  |
| 52 | Dong 2018 |  |  |  | X |  |  |  |  |  |
| 53 | Fei 2018 |  |  |  |  | X | X |  |  |  |
| 54 | Han 2018 |  |  | X |  | X |  |  |  |  |
| 55 | Aflatoonian 2020 |  | X | X |  | X |  |  |  |  |
| 56 | Diaz 2020 |  |  |  |  |  | X |  |  |  |
| 57 | Garcia -tejedor 2020 | X |  | X |  | X |  |  |  |  |
| 58 | Miquel 2020 |  | X | X |  | X |  |  |  |  |
| 59 | Alborzi 2021 | X | X | X |  |  |  |  |  |  |
| 60 | Huang 2021 |  | X | X |  |  |  |  |  |  |
| 61 | Koo 2021 | X |  | X |  | X |  |  |  |  |
| 62 | Martinez-garcia 2021 | X |  | X |  |  |  |  |  |  |
| 63 | Ghasemi Tehrani 2022 |  | X |  |  |  |  |  |  |  |
| 64 | Lee 2022 |  |  | X |  |  |  |  |  |  |
| 65 | Meng 2022 |  |  | X |  |  |  |  |  |  |
| 66 | Anvari Aliabad 2023 |  | X |  |  |  |  |  |  |  |
| 67 | Vaduva 2023 |  | X |  |  |  |  |  |  |  |
